# Supplementary material for: Structural Variants of Dermatan Sulfate Can Affect the Expression of Proteins Involved in Breast Cancer Cell Survival
Source: Cells. 2025 Oct 11;14(20):1581. doi: 10.3390/cells14201581 (PMC12564227; doi:10.3390/cells14201581)
Supplement: Supplementary file 1 [file cells-14-01581-s001.zip › cells-3861696-supplementary.pdf]

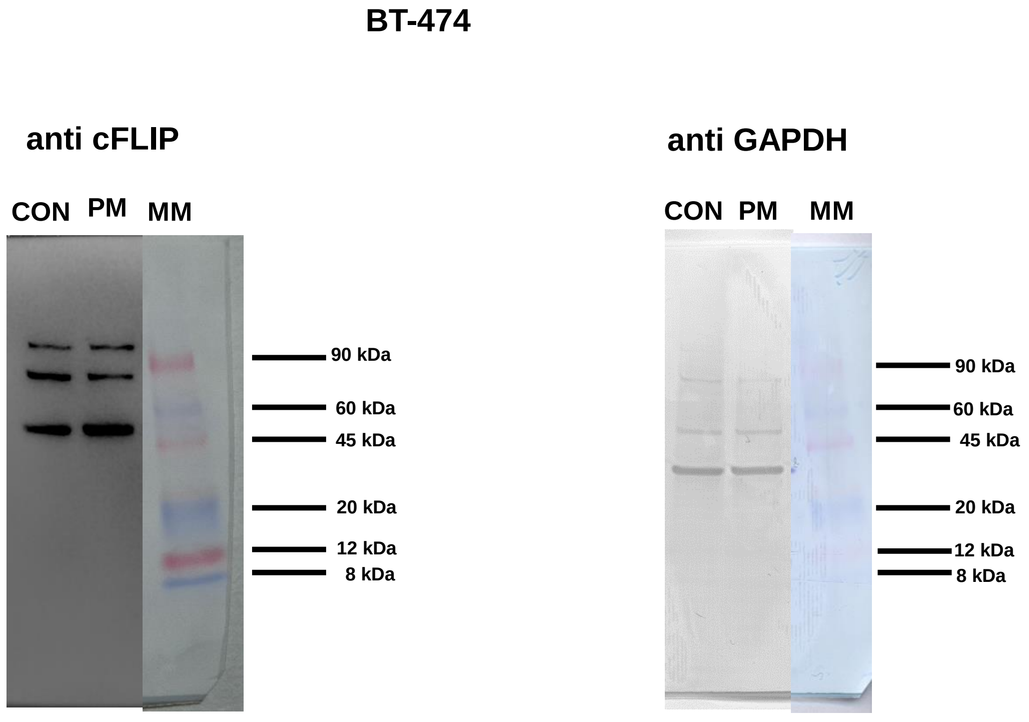

Figure S1. The full-size blots, showing the stimulating effect of the PM variant on the cFLIP(L) level in BT-474 breast cancer cells that were cultured in the presence of this glycan for three hours. MM – molecular markers.

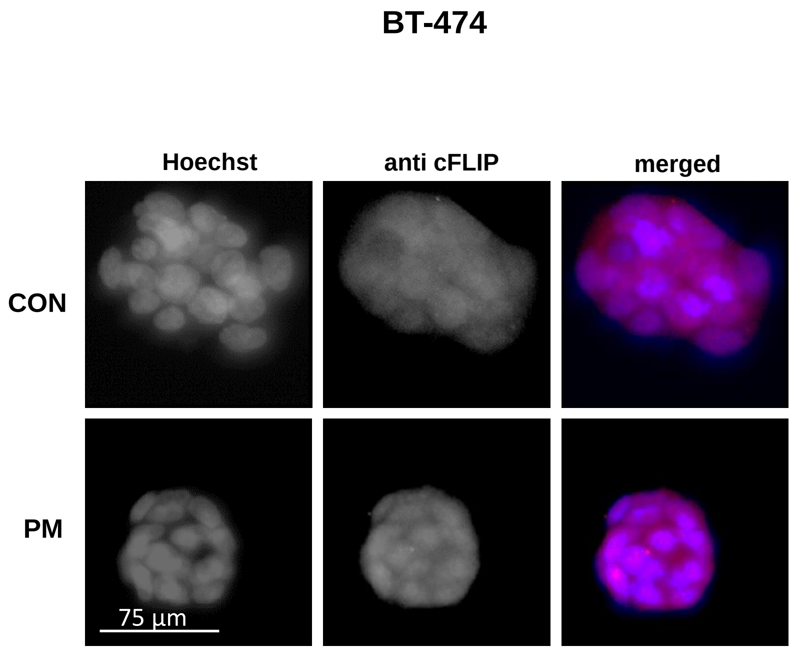

Figure S2. Representative images, showing the PM-dependent effect on the nuclear distribution of cFLIP in BT-474 breast cancer cells.

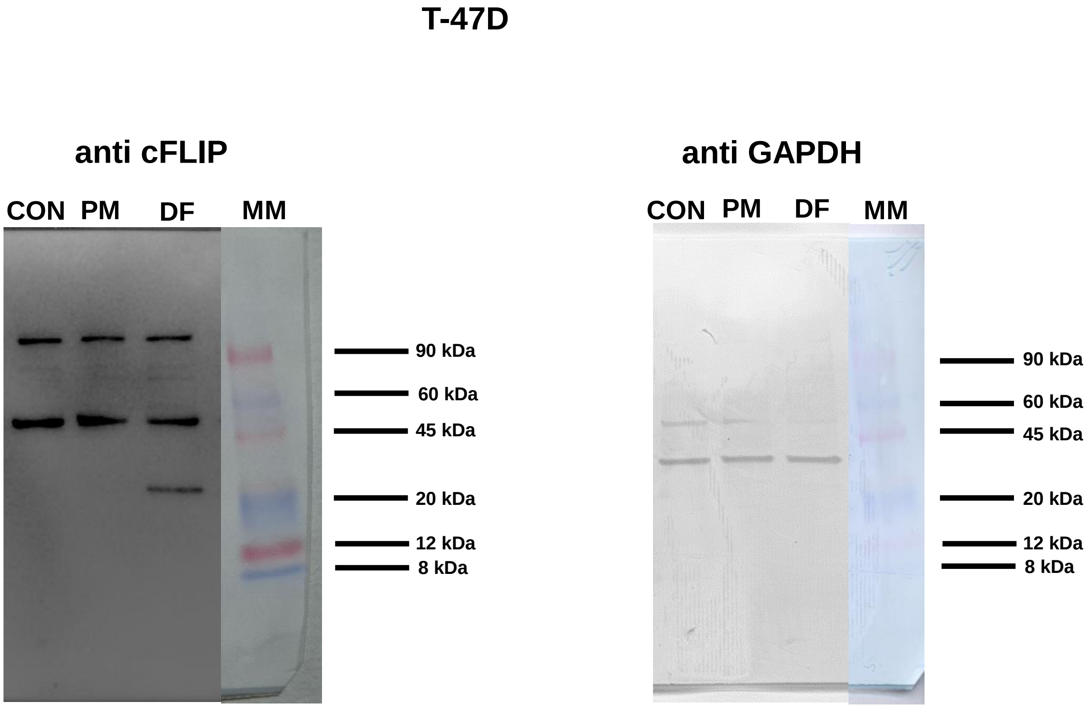

Figure S3. The original blots, illustrating the effects of the tested DS variants, i.e. PM and DF, on the cFLIP expression in T-47D breast cancer cells that were grown in the presence of these glycans for three hours. MM – molecular markers.

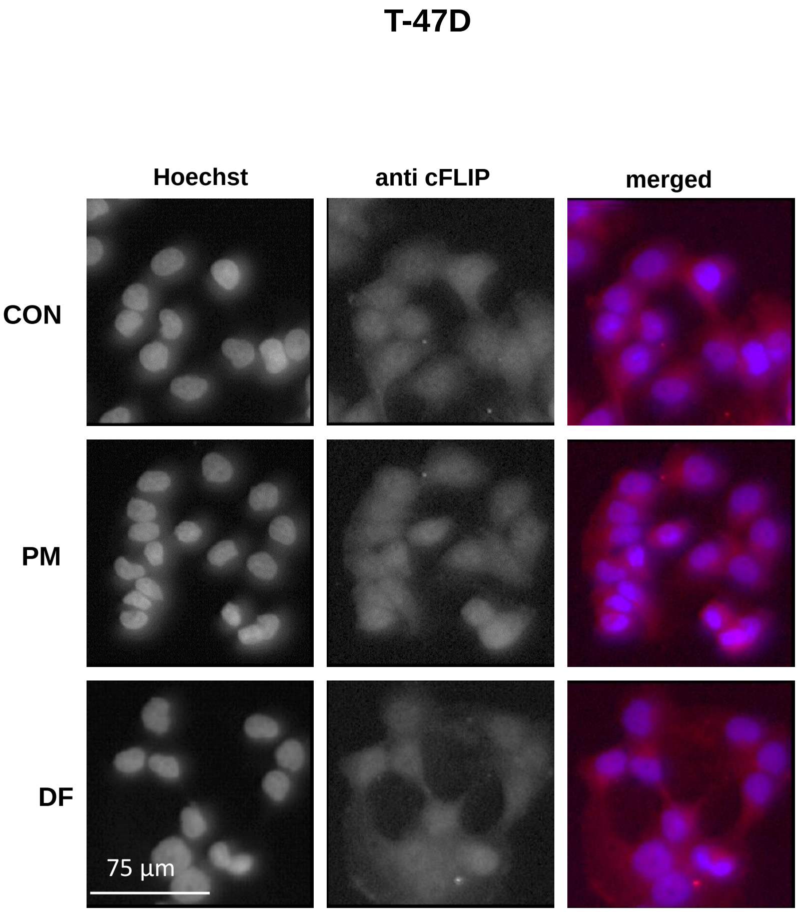

Figure S4. Representative images, showing the PM- and DF-dependent effect on the nuclear distribution of cFLIP in T-47D breast cancer cell line.

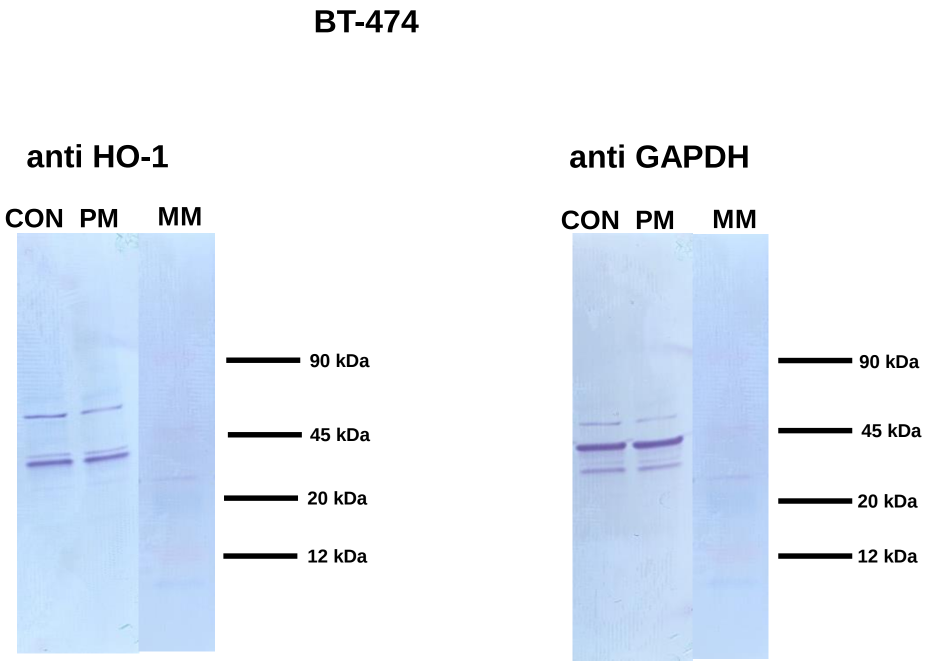

Figure S5. The full-size blots, showing the influence of the PM variant on HO-1 level in BT-474 breast cancer cells that were exposed to this glycan for two hour. MM – molecular markers.

BT-474

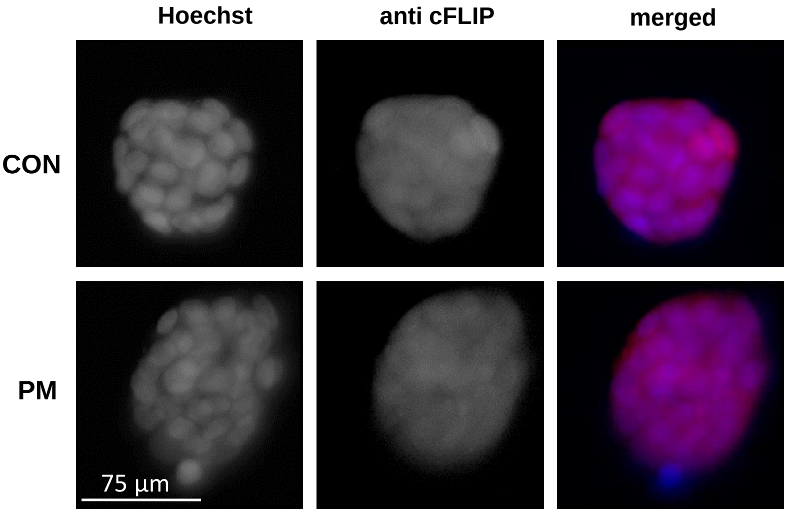

Figure S6. Representative images, showing the PM-dependent effect on the nuclear distribution of HO-1 in BT-474 breast cancer cells.

T-47D

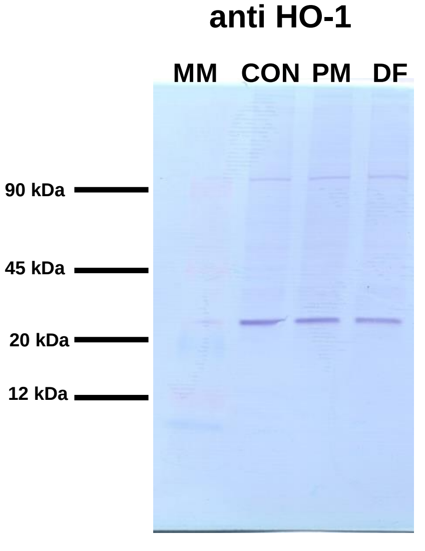

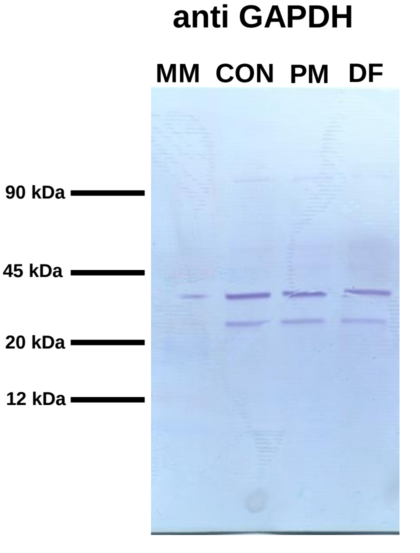

Figure S7. The original blots, showing downregulation of HO-1 in T-47D breast cancer cells that were cultured in the presence of PM or DF for two hours. MM – molecular markers.

T-47D

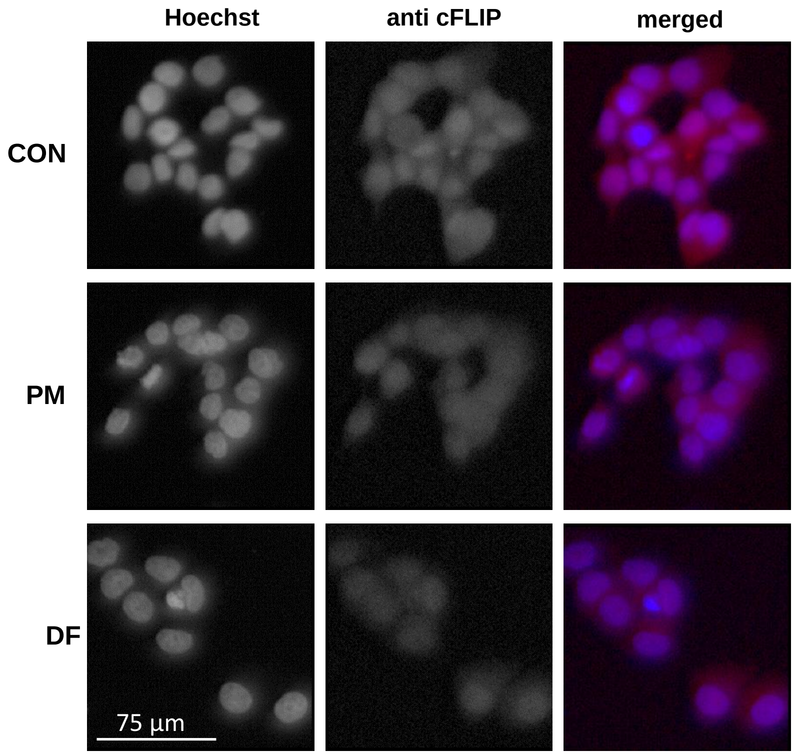

Figure S8. Representative images, showing the PM- and DF-dependent effect on the nuclear localization of HO-1 in T-47D breast cancer cells.

BT-474

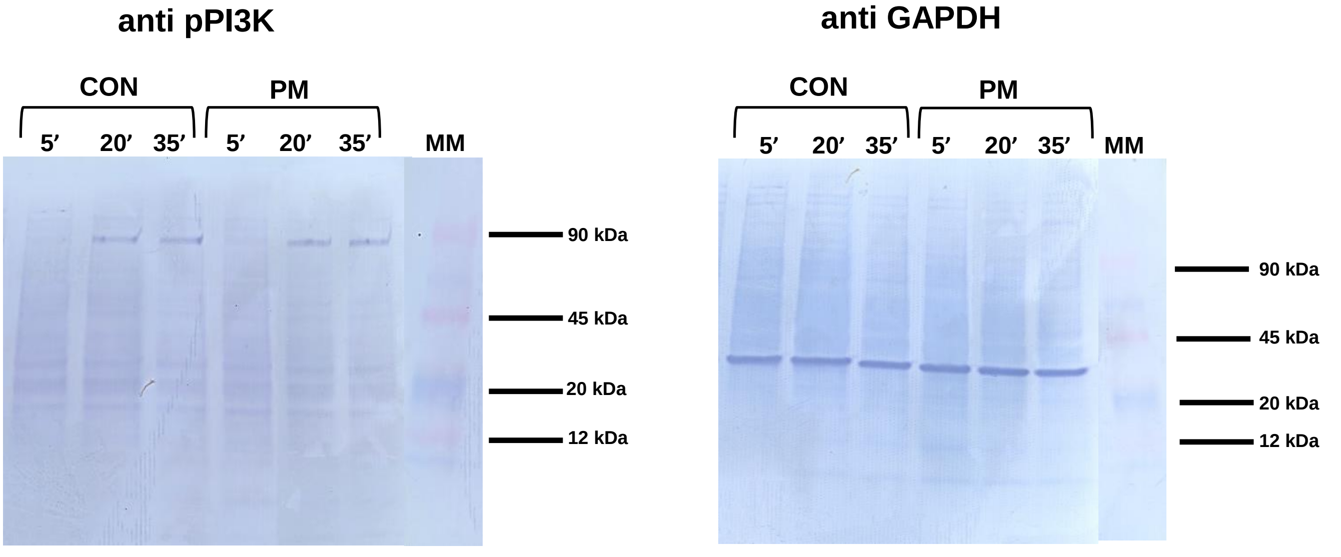

Figure S9. The full-size blots, showing the kinetics of the PM-induced activation of PI3K in BT-474 breast cancer cells. MM – molecular markers.

BT-474

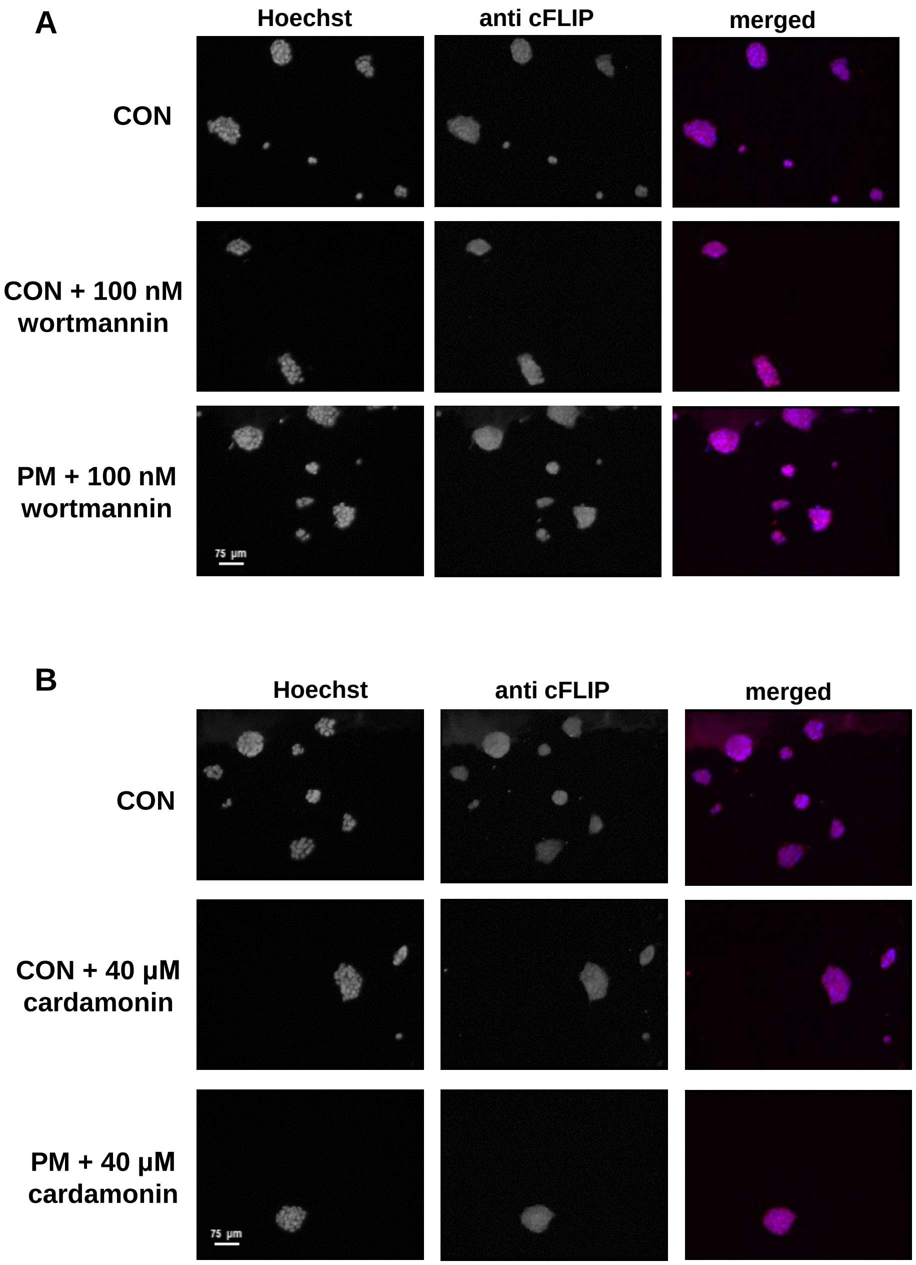

Figure S10. Representative images, illustrating the effect of wortmannin (an inhibitor of PI3K activity) (A) or cardamonin (an inhibitor of NFκB activity) (B) on the PM-dependent upregulation of cFLIP in BT-474 breast cancer cells.

BT-474

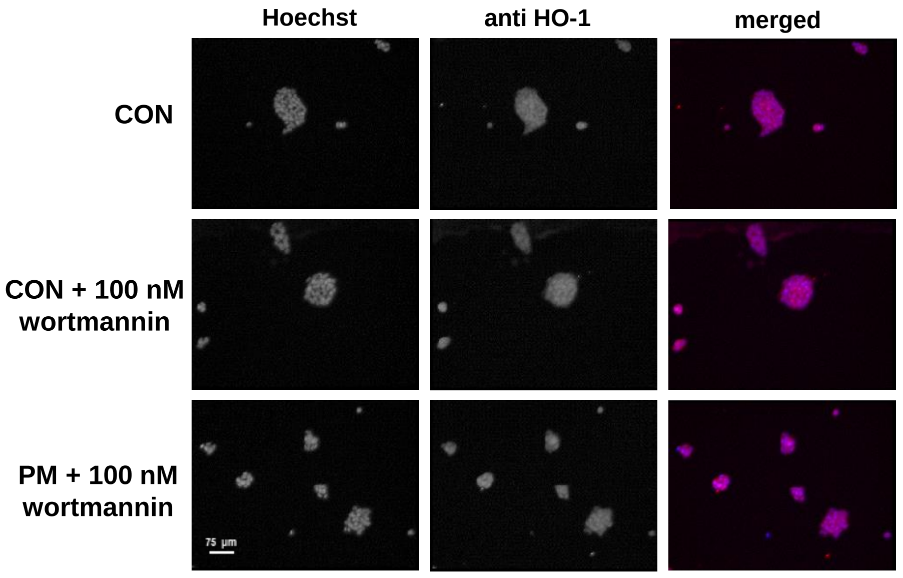

Figure S11. Representative images, showing the effect of wortmannin on HO-1 level in BT-474 cancer cells that were exposed to the PM variant for two hours.

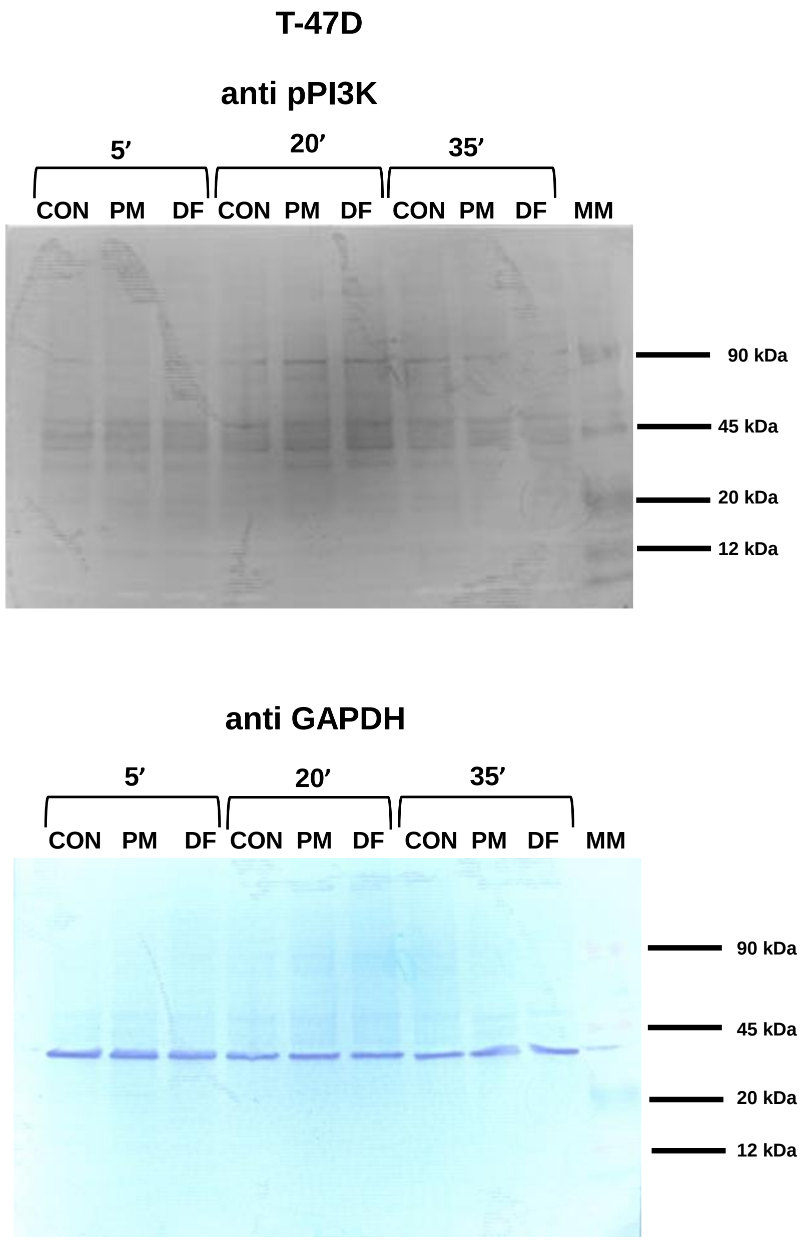

Figure S12. The original blots, showing the kinetics of the PM- or DF-induced activation of PI3K in T-47D breast cancer cells. MM – molecular markers.

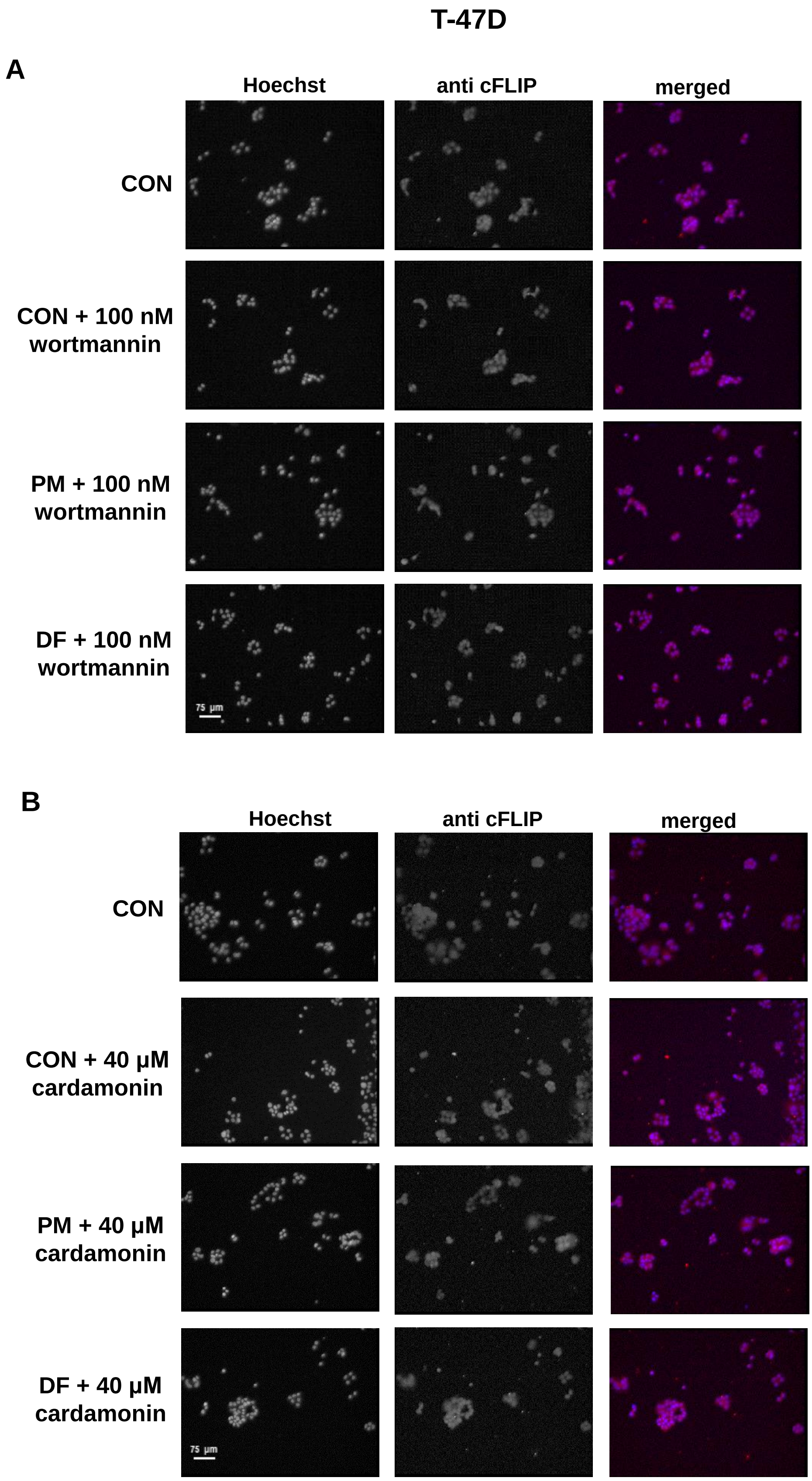

Figure S13. Representative images, showing the effect of wortmannin (A) or cardamonin (B) on the DS variant-triggered upregulation of cFLIP in T-47D breast cancer cells.

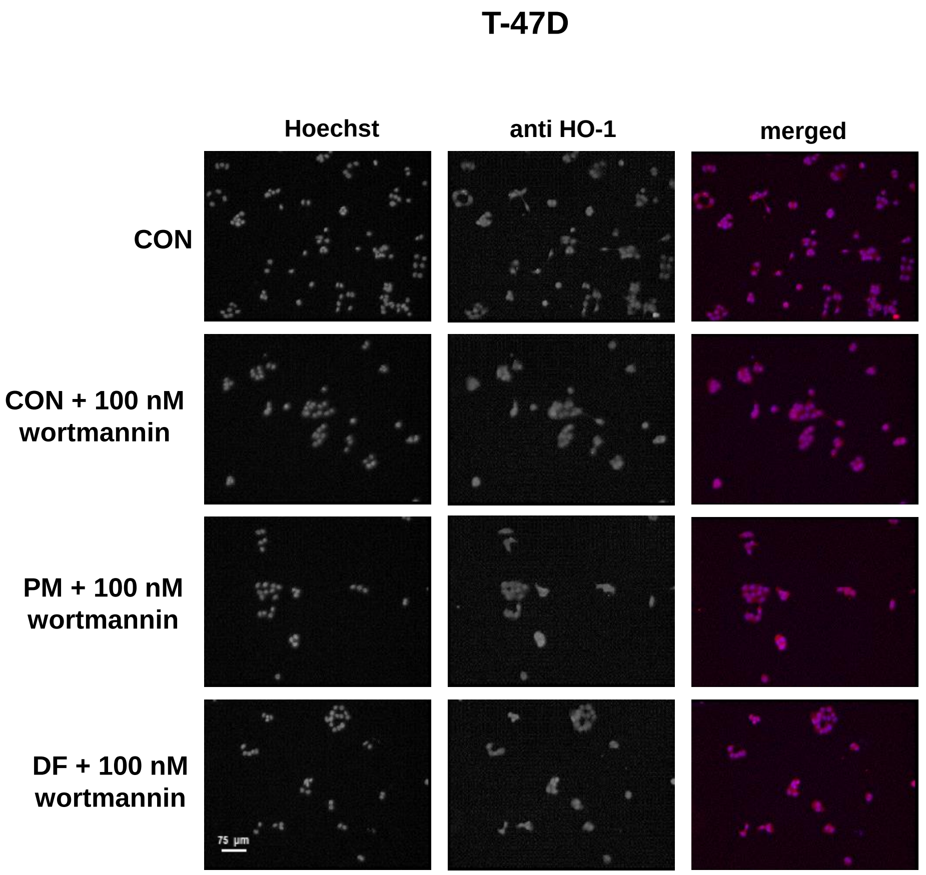

Figure S14. Representative images, illustrating the impact of wortmannin on the PM or DF-induced downregulation of HO-1 in T-47D breast cancer cells.
